# Microwave Absorption Properties of Carbon Black-Carbonyl Iron/Polylactic Acid Composite Filament for Fused Deposition Modeling

**DOI:** 10.3390/ma15155455

**Published:** 2022-08-08

**Authors:** Fei Wang, Qianfeng Zhou, Zhe Zhang, Yonghua Gu, Jiliang Zhang, Kaiyong Jiang

**Affiliations:** 1 Fujian Key Laboratory of Special Energy Manufacturing, Huaqiao University, Xiamen 361021, China; 2 Xiamen Key Laboratory of Digital Vision Measurement, Huaqiao University, Xiamen 361021, China

**Keywords:** absorbing materials, dielectric loss, magnetic loss, 3D printing, FDM filament

## Abstract

A single microwave absorbent and simple coating structure cannot meet the increasing requirements for broadband and strong absorption. Three-dimensional printing is an effective way to prepare multi-component complex structure metamaterial absorbers, and the key is to prepare raw materials with excellent absorption properties, suitable for 3D printing. In this paper, CB-CIP/PLA composite filament was prepared via a high-energy mixer and twin-screw extruder by compounding the dielectric loss material carbon black (CB) and the magnetic loss material carbonyl iron powder (CIP) with polylactic acid (PLA) as the matrix. The coaxial ring test piece was printed by FDM technology, and the microstructure of the composites was observed and analyzed by SEM. Meanwhile, the electromagnetic parameters of the composites were examined by a vector network analyzer, mainly studying the influence of the CB and CIP content and thickness on the microwave absorbing properties of the composite material. The results show that when the CB content is 20% and the CIP content is 30%, the CB-CIP/PLA composite has excellent microwave absorption and broad bandwidth. When the matching thickness is 1.6 mm, the minimum reflection loss (*RL*) reaches −51.10 dB; when the thickness is 1.7 mm, the effective absorption bandwidth (*RL* < −10 dB) is 5.04 GHz (12.96–18 GHz), nearly covering the whole Ku band. This work provides an efficient formulation and process to prepare an absorbing composite filament for FDM.

## 1. Introduction

Microwave absorbing materials (MAMs) are an effective means of preventing electromagnetic pollution and protecting human health. Electromagnetic stealth technology has always been a research hotspot in the military industry, and MAMs are a key factor in this technology [1]. Therefore, MAMs have important research value and wide application prospects in civil and military fields [2]. At present, the development of thin, high-efficiency and broadband-absorbing materials has attracted much attention [3,4,5].

According to the loss mechanism, the absorbing agent is divided into the following three types: the dielectric type, the magnetic type and the resistive type. As a magnetic loss agent, carbonyl iron powder (CIP) has the advantages of low cost, high saturation magnetization and magnetic permeability and has attracted the attention of researchers. However, its high density makes it difficult to meet the low weight requirements [6,7]. Carbon-based materials, as dielectric loss agents, have the advantages of low density, large specific surface area, high thermal stability and great electrical conductivity.

As users have increasing requirements for the comprehensive properties of absorbing materials, single iron-based or carbon-based absorbing agents have been unable to meet the needs. Performance complementarity can be achieved by combining CIP with carbon-based material. This can not only reduce the density but also provide it with both dielectric and magnetic losses, which can then be lightweight with broadband and strong absorption. Liu et al. [8] obtained C@CIPs composites by mechanical milling and other processes using MWCNTs and CIPs with silicone rubber as the matrix. Since the MWCNTs were coated on the surface of the CIPs, the electromagnetic parameters of the material were improved. When the thickness was 0.6 mm, the content of the MWCNTs was 5%, and the content of the CIPs was 45%; it had a certain wave absorbing performance, but its reflection loss was only −5.48 dB. Qing et al. [9] filled different mass fractions (1–6%) of carbon fiber (CF) and 65 wt.% carbonyl iron (CI) into epoxy/silicone resin to prepare absorbing coatings. The addition of CF increased the complex permittivity of the material but did not affect the complex permeability, and the combination of the two improved the absorption bandwidth. As the CF content increased or the thickness of the composite coating increased, the minimum reflection loss and the absorption band shifted to the lower frequency. When the CF content was 2%, and the coating thickness was 1 mm, the reflection loss was lower than −10 dB in the frequency range of 8–18 GHz. Ye et al. [10] used polylactic acid (PLA) as a matrix to composite 20% carbonyl iron powder (CIP) with different mass fractions (0–5%) of graphene (RGO) to prepare an RGO–CIP/PLA composite. When the thickness was 3 mm, and the RGO content was 4%, the reflection loss value reached −27.25 dB, and the effective absorption bandwidth was 2.922 GHz. At this time, the composite material had the best wave absorption performance. The aforementioned research tried to improve the microwave absorbing performance through the existence of synergy between CIP and carbon-based absorbing agents. However, these absorbing materials remain in the form of coatings.

Currently, metamaterial absorbers with complex structures show better absorbing properties than coatings. Three-dimensional (3D) printing technology has a natural advantage in shaping such metamaterial absorbers. The key problem is how to prepare raw materials with excellent absorbing properties that cater to a 3D printing process.

In this paper, PLA, CB and CIP serve as the matrix, dielectric loss absorbent and magnetic loss absorbent. The CB-CIP/PLA composite filament for Fused Deposition Modeling (FDM) was prepared by high-energy mixing and twin-screw melt extrusion process. The influence of the CB and CIP content and thickness on the absorbing properties was studied. The preparation of the filament is simple and inexpensive. Through the FDM process, the filament can be used for printing a metamaterial absorber, realizing the integration of material, design and manufacturing, which has broad application prospects.

## 2. Materials and Methods

### 2.1. Materials

PLA powder, 4032D, with a true density of 1.25 g/cm^3^, was provided by Huachuang Plastic Technology Co., Ltd., Dongguan, China. CB, with a specific surface area of 55–70 m^2^/g, the resistivity of 2.5 Ω·m, the apparent density of 0.1176 g/cm^3^ and a true density of 1.8 g/cm^3^, was provided by Zhengyuan Technology Co., Ltd., Tianjin, China. CIP, with an apparent density of 3.2–3.5 g/cm^3^ and true density of 4.0 g/cm^3^, was provided by Lebo Metal Material Technology Co., Ltd., Hebei, China. Aluminate coupling agent (AL) was provided by Feiteng Chemical Co., Ltd., Nanjing, China.

### 2.2. CB-CIP/PLA Composites Preparation

The CB was ground and then placed in a vacuum drying oven (VDM50, IRM Technology GmbH, Germany) with PLA and dried at 80 °C for 6 h to eliminate excess moisture. The aluminate coupling agent with a mass fraction of 1.5% was added to PLA, then a certain proportion of CB and CIP (wt.%, relative to the PLA) were added, respectively, and finally put into a dual-motion high-energy mixer (JHT-5, Gold Co-Powder Technology Co., Ltd., Zhengzhou, China). The rotating speed of the barrel and the blade was set to 28 r/min and 17 r/min, respectively, and the mixing time was 1 h to ensure uniform mixing.

**Figure 1:**
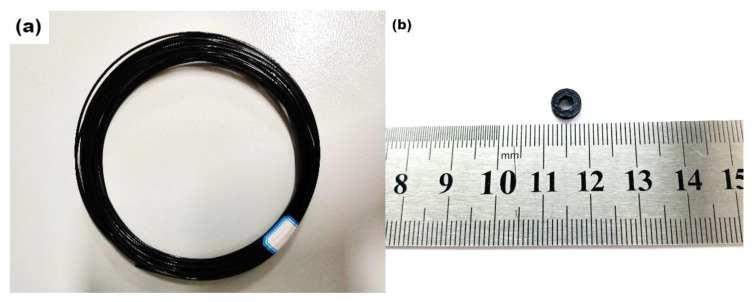
(**a**) CB-CIP/PLA composite filament; (**b**) absorption test coaxial ring.

The CB-CIP/PLA composite filament (as illustrated in Figure 1a) was obtained by adding the uniformly mixed composite powder into a twin-screw extruder (SJZS-10B, Ruiming Experimental Instrument Manufacturing Co., Ltd., Wuhan, China) after preheating, followed by melting blending, extrusion molding, air cooling and winding with a traction coiler. The extruder parameters are set as follows: zone 1 temperature 145 °C, zone 2 temperature 165 °C, zone 3 temperature 175 °C and head temperature 175 °C; the screw speed was 18 r/min, and the filament diameter was controlled at 1.75 ± 0.05 mm. 

By using a desktop 3D printer (CR-5, Chuangxiang 3D Technology Co., Ltd., Shenzhen, China), the self-made CB-CIP/PLA composite filament was printed out as the coaxial ring test pieces. The outer diameter of the coaxial ring was 7 mm, the inner diameter was 3 mm and the thickness was 2 mm (as illustrated in Figure 1b). The pre-built model (3D printing compatible STL file) was imported into Modellight software, then set the parameters and sliced, which were saved in Gcode format. Modellight slicing software was used to slice the pre-built model and set the parameters, which were transferred to a 3D compatible STL file. The printing parameters were: nozzle aperture 0.4 mm, layer height 0.1 mm, printing speed 40 mm/s, nozzle temperature 210 °C, bed temperature 40 °C and filling density 100%.

### 2.3. Experimental Design

According to previous studies, a too-low content of CB and CIP makes the absorbing effect poor, while a too-high content makes it difficult to extrude. Therefore, the CB content was selected as 5–20%, and the CIP content was selected as 10–50%. Meanwhile, the variation in CB and CIP content results in the variation in electromagnetic parameters and impedance matching, which affects the absorption properties of the materials. Therefore, a single-factor experiment was designed to investigate the influence of CB and CIP on absorption performance and determine the optimal ratio. The experimental scheme is shown in Table 1.

### 2.4. Test and Characterization

The density of these filaments was measured by the Archimedes method. A scanning electron microscope (JSM-IT500, JEOL Ltd., Tokyo, Japan) was used to analyze the morphologies and structures of the composites. The electromagnetic parameters of the CB-CIP/PLA composites were tested in the range of 2–18 GHz by means of a vector network analyzer (Keysight Agilent E5071C) by coaxial transmission line method.

## 3. Results and Discussion

### 3.1. Micromorphology of Absorbent

**Figure 2:**
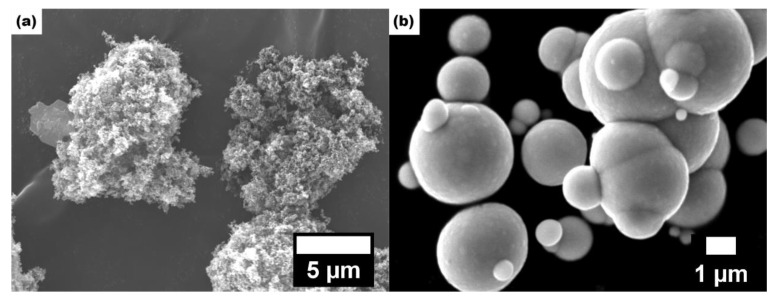
SEM micrographs of absorbent: (**a**) CB; (**b**) CIP.

Figure 2a shows the microscopic morphology of the CB particles under SEM (5000×). The CB particles had a large specific surface area and surface energy with nanometer diameter; thus, agglomeration occurred. Agglomeration contributes to the formation of conductive networks, thus increasing the conductivity and dielectric constant of the material. Figure 2b shows the microscopic morphology of the CIP particles under SEM (10,000×). It can be seen that the surface of the CIP particles was spherical and smooth, with a particle size of about 1–5 μm.

### 3.2. Influence of Absorbent Content on the Electromagnetic Parameters of the Composites

The electromagnetic parameters of materials are important factors affecting their absorption of electromagnetic waves. Among them, the complex permittivity ($\varepsilon =\varepsilon^{\prime }-j\varepsilon^{\prime \prime }$) is determined by the electrical properties of the material, while the complex permeability ($\mu =\mu^{\prime }-j\mu^{\prime }$) is determined by the magnetic properties. Both real parts contribute to electromagnetic energy storage, while both imaginary parts contribute to dissipation [11]. Dielectric loss tangent ($\tan\delta_{\varepsilon }=\varepsilon^{\prime \prime }/\varepsilon^{\prime }$) and magnetic loss tangent ($\tan\delta_{\mu }=\mu^{\prime \prime }/\mu^{\prime }$) are two loss factors to measure the dissipative ability of materials [12,13].

**Figure 3:**
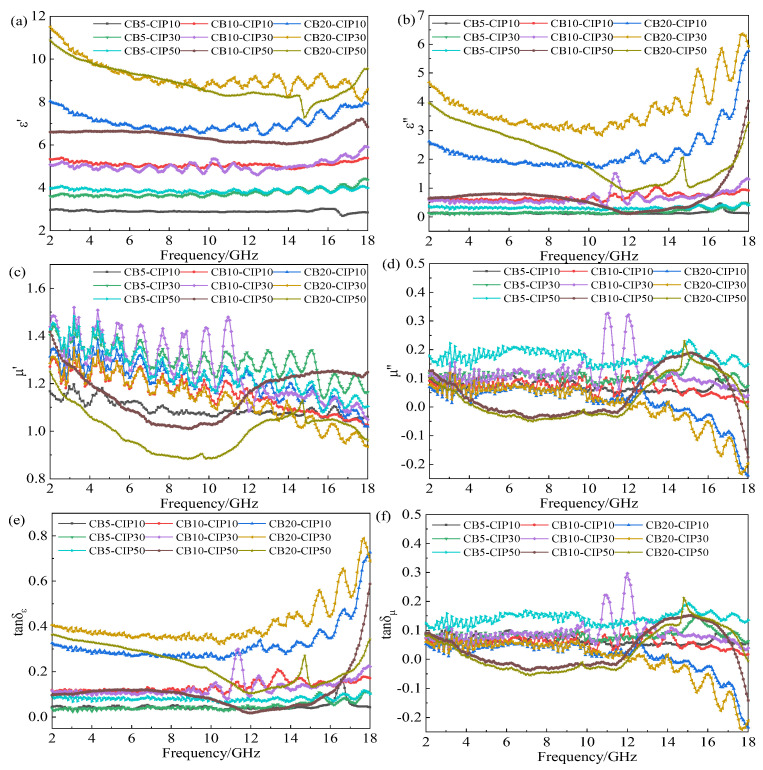
Electromagnetic parameters of different CB-CIP/PLA composites: (**a**) real part of complex permittivity; (**b**) imaginary part of complex permittivity; (**c**) real part of complex permeability; (**d**) imaginary part of complex permeability; (**e**) dielectric loss tangent; (**f**) magnetic loss tangent.

Figure 3 shows the electromagnetic parameters of the different CB–CIP/PLA composites (“CB5-CIP10” in the figure means “CB content 5%—CIP content 10%”, which is the same below).

Firstly, the influence of the CB content on the electromagnetic parameters of the composites was analyzed under the condition of a constant CIP content. From Figure 3a,b,e, it can be found that when the CB content was 5%, the real and imaginary parts of the complex permittivity tended to be constant in the range of 2–18 GHz, with slight fluctuations, and the value was relatively small. The dielectric loss tangent did not exceed 0.1, indicating poor dielectric loss ability. With the increase in CB content, both the real and imaginary parts of the complex permittivity increased to a certain extent but slowly. However, when the CB content increased to 20%, the real and imaginary parts of the complex permittivity increased significantly, and the dielectric loss ability was obviously enhanced, which may be caused by the conductive loss. When the CB content reached a certain threshold, the conductive network in the composite was gradually completed and continued to increase, which caused the conductivity of the material to increase, and the conductive loss was significantly enhanced. In addition, the changing trend of the dielectric loss tangent and imaginary part of the complex permittivity was basically the same, and both had certain fluctuations. From Figure 3c,d,f, it can be seen that with the increase in CB content, the magnetic permeability of the composite material changes obviously, because CB is a non-magnetic material. However, when the CB content was 20%, both the real and imaginary parts of the complex permeability decreased to a certain extent.

Then, the influence of the CIP content on the electromagnetic parameters of the composites was analyzed under the condition of a constant CB content. From Figure 3a–d, it can be observed that when the CB content was low, with the increase in the CIP content, the real part of the complex permittivity increased to a certain extent, but the imaginary part nearly did not change. When the CB content reached 20%, the real and imaginary parts of the complex permittivity first increased and then decreased with the increase in the CIP content. When the CB content was 20% and the CIP content was 50%, the complex permittivity and complex permeability of the composites both decreased significantly. This is due to the fact that the content of the absorbent was too high, resulting in agglomeration in some areas, which seriously affected the absorbing properties.

Finally, it is remarkable that the $\mu^{\prime \prime }$ of the three groups of composites CB20-CIP10, CB20-CIP30 and CB20-CIP50 were negative in the high-frequency regions above 12 GHz, which is attributed to the magnetic energy radiating outward from the sample. According to Maxwell’s equations, permittivity and permeability are interrelated, especially the movement of electric charge produces a magnetic field; therefore, part of the electric field energy is converted into magnetic energy and radiates outward, resulting in a negative $\mu^{\prime \prime }$ [14,15].

As can be observed from Figure 3e,f, the loss of electromagnetic waves in materials was mainly dielectric loss, followed by magnetic loss.

### 3.3. Influence of Absorbent Content on Absorbing Properties Components

Generally, the absorbing properties of composites could be assessed by reflection loss (*RL*) and effective absorption bandwidth (EAB, the corresponding bandwidth of *RL* < −10 dB) [16,17,18]. *RL* is generally expressed as a negative value; the smaller the loss by reflection, the larger the loss by absorption. According to the value of *RL*, the percentage of absorption is shown in Table 2 [19,20].

According to the transmit-line theory, *RL* is usually calculated from Equations (1) and (2) [21]:

(1) $$RL=20\mathrm{lg}\left|\frac{Z_{in}-Z_{0}}{Z_{in}+Z_{0}}\right|$$

(2) $$Z_{in}=Z_{0}\sqrt{\frac{\mu_{r}}{\varepsilon_{r}}}\tanh\left[j\left(\frac{2\pi fd}{c}\right)\sqrt{\mu_{r}\varepsilon_{r}}\right]$$

where $Z_{0}=\sqrt{\frac{\mu_{r}}{\varepsilon_{r}}}\approx 377\Omega$ is the impedance of free space, $Z_{in}$ is the input impedance of the absorber, $\varepsilon_{r}$ is the complex permittivity, $\mu_{r}$ is the complex permeability, h is Planck constant, j is the imaginary unit, f is the frequency of the incident microwave, d is the thickness of the absorbing material and c is the velocity of light.

**Figure 4:**
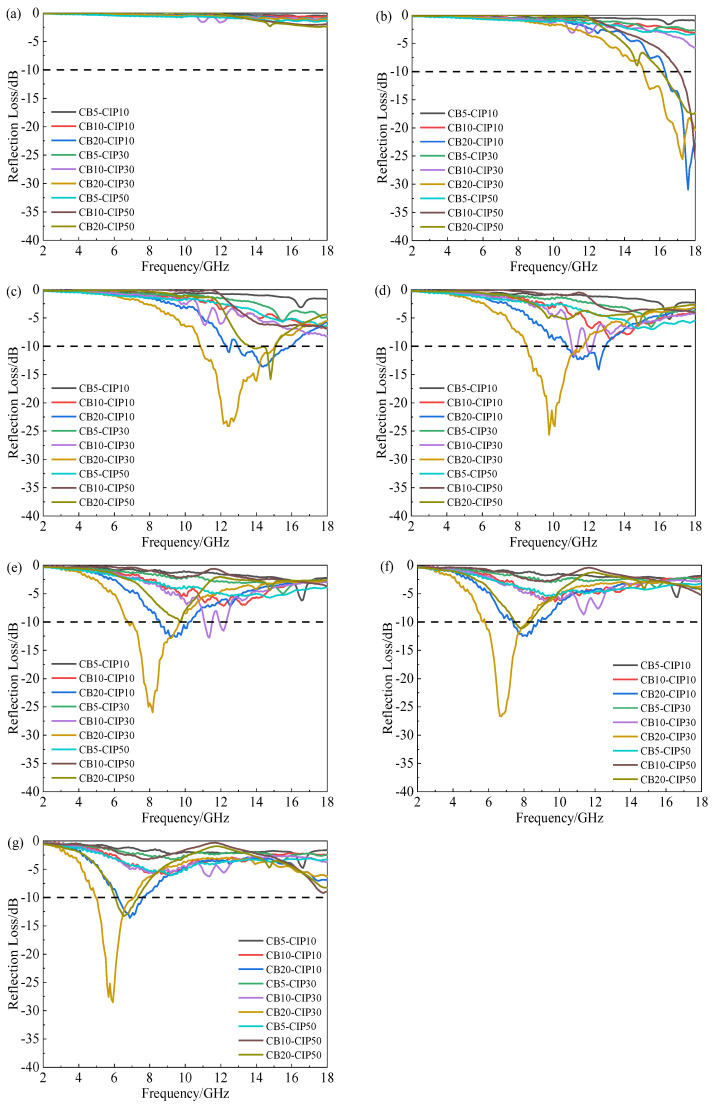
*RL* diagram of the CB-CIP/PLA composites with different thicknesses: (**a**) 1 mm; (**b**) 1.5 mm; (**c**) 2.0 mm; (**d**) 2.5 mm; (**e**) 3.0 mm; (**f**) 3.5 mm; (**g**) 4.0 mm.

Figure 4 shows the influence of the CB and CIP contents on the *RL* of the composite materials at different thicknesses. It can be seen that the minimum *RL* of each component of the composite shifted to the low-frequency band as the thickness of the absorber layer increased. In Figure 4a, when the thickness was 1 mm, the *RL* value of each group did not reach −10 dB, indicating that the overall absorbing performance was poor. When the thickness was increased to 1.5 mm, the CB20-CIP10 composite showed a peak at high frequency (16–18 GHz), but the effective absorption bandwidth was only 1.68 GHz. As the thickness increased to 2.0 mm, the peak value of *RL* gradually shifted to the left, and the effective absorption bandwidth increased obviously. The CB20-CIP10, CB20-CIP30 and CB20-CIP50 composite had better absorbing effects, among which the CB20-CIP30 composite had the widest effective absorption bandwidth. It reached 4.08 GHz (10.88–14.96 GHz), and the minimum *RL* was about −25 dB. However, as the thickness continued to increase, the effective absorption bandwidth became narrower, and the absorbing effect gradually weakened.

**Figure 5:**
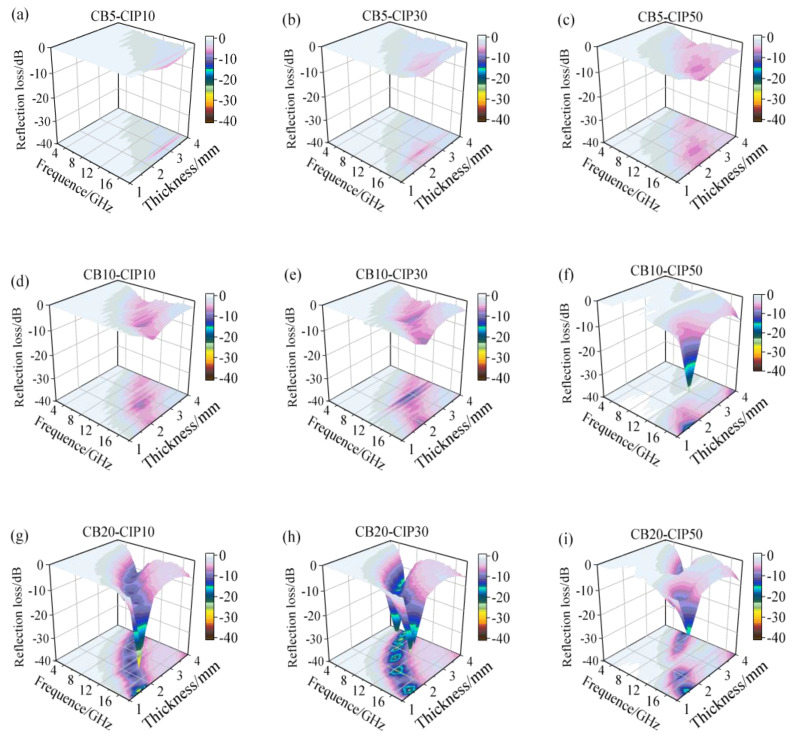
Three-dimensional diagrams of *RL* of the composites with different compositions: (**a**) CB5-CIP10; (**b**) CB5-CIP30; (**c**) CB5-CIP50; (**d**) CB10-CIP10; (**e**) CB10-CIP30; (**f**) CB10-CIP50; (**g**) CB20-CIP10; (**h**) CB20-CIP30; (**i**) CB20-CIP50.

Figure 5 shows the three-dimensional diagram of the reflection loss and thickness frequency of the composites with different components. It can be observed that the absorption effect of the three groups of composite materials with a CB content of 5% was poor. When the thickness was 1.0–2.0 mm, the CB content was 20%, and the CIP content was 10% and 30%, the absorbing performance was improved. By observing the 3D projection map, it can be noticed that the minimum *RL* gradually shifted to a low frequency as the thickness increased. Similarly, as the thickness increases, the EAB with *RL* less than −10 dB shifts to the lower frequency band [22]. The shift of the minimum *RL* could be explained by the quarter-wavelength cancellation theory [23]. When the thickness of absorbing material is precisely 1/4 of the incident wave length, the phase difference between the incident and reflected electromagnetic waves is 180°. The incident wave and reflected wave cancel each other due to opposite phases and equal wavelengths. By adjusting the thickness of the absorption layer, electromagnetic waves in a specific frequency band could be effectively absorbed.

### 3.4. Absorbing Properties of the CB20-CIP30 Composite

**Figure 6:**
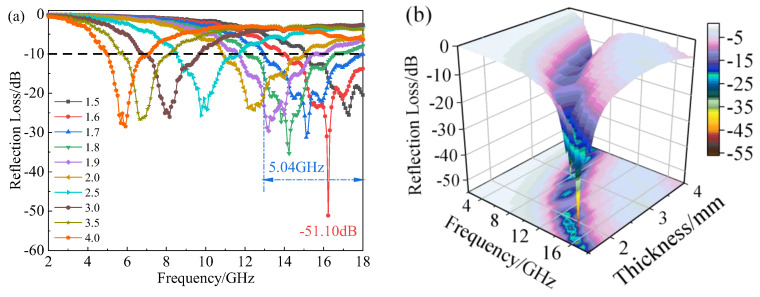
(**a**) *RL* diagram of the CB20-CIP30 composite with different thicknesses; (**b**) Three-dimensional diagrams of *RL* of the CB20-CIP30 composite with different thicknesses.

**Figure 7:**
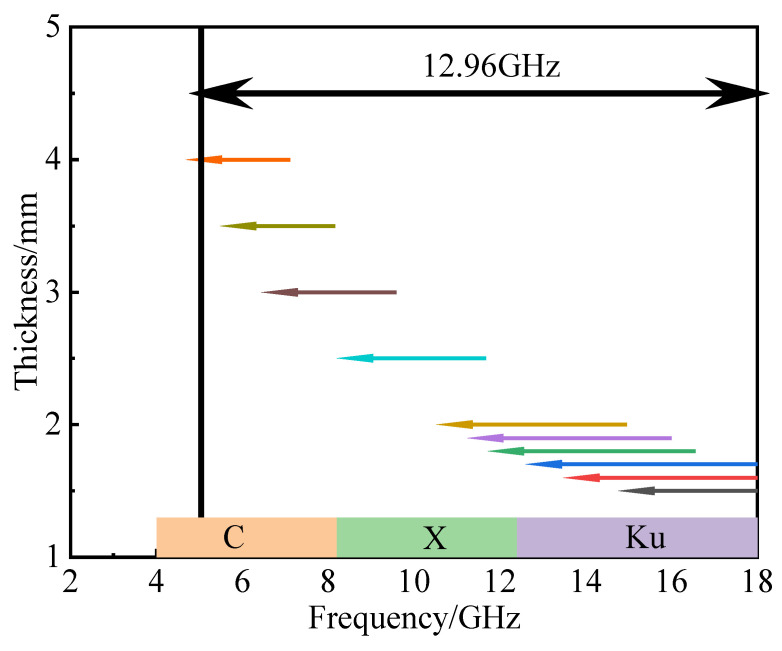
Effective absorption bandwidths of the CB20-CIP30 composite at different thicknesses.

Based on the above analysis and comparison, the material properties in the preparation process were integrated. Finally, 20% CB and 30% CIP were determined as the best components, and their absorbing properties were analyzed in detail. Figure 6 shows the *RL* diagram and corresponding three-dimensional diagram of the material at a thickness of 1.5–4.0 mm. It can be found that the composite material achieved an effective electromagnetic wave absorption at 1.5–4.0 mm. When the thickness was 1.6 mm, the minimum *RL* value was −51.10 dB (at 16.24 GHz). At this time, the microwave absorption rate was close to 100%. When the thickness was 1.7 mm, the effective absorption bandwidth reached 5.04 GHz (12.96–18 GHz), covering 90% of the Ku-band (12.4–18 GHz). The effective absorption bandwidth of the CB20-CIP30 composite at different thicknesses is shown in Figure 7. Within the thickness range of 1.5–4.0 mm, the effective absorption bandwidth of the CB20-CIP30 composite reached 12.96 GHz (5.04–18 GHz), which could completely cover the Ku-band, X-band (8.2–12.4 GHz) and 75% of the C-band (4–8.2 GHz).

The absorbing properties of the CB20-CIP30 composite and other CIP composite absorbing materials were further compared, as shown in Table 3. The comparison found that, except for references [24], the absorption intensity of the CB20–CIP30 composite was the highest, and the minimum *RL* reached −51.10 dB. At this time, the microwave absorption rate was close to 100%, which can meet the requirements of the strong absorption of the absorbing materials. Compared with references [25,26,27,28], the effective absorption bandwidth of the CB20-CIP30 composite needs to be improved, but the CB20-CIP30 composite can achieve effective electromagnetic wave absorption at a lower thickness, which can meet the lightweight requirements of absorbing materials, and solve the problems of the complex process, environmental pollution, low efficiency and high cost in chemical preparation. It is worth noting that the PLA matrix used in this paper is a degradable and environmentally friendly material that meets the requirements of sustainable development. Finally, the CB20-CIP30 composite prepared in this paper can directly use FDM technology to form complex and fine absorbing structures to realize the integration of material, design and manufacturing.

### 3.5. The Synergy of the Multiple Loss Mechanism of the CB20-CIP30 Composite

**Figure 8:**
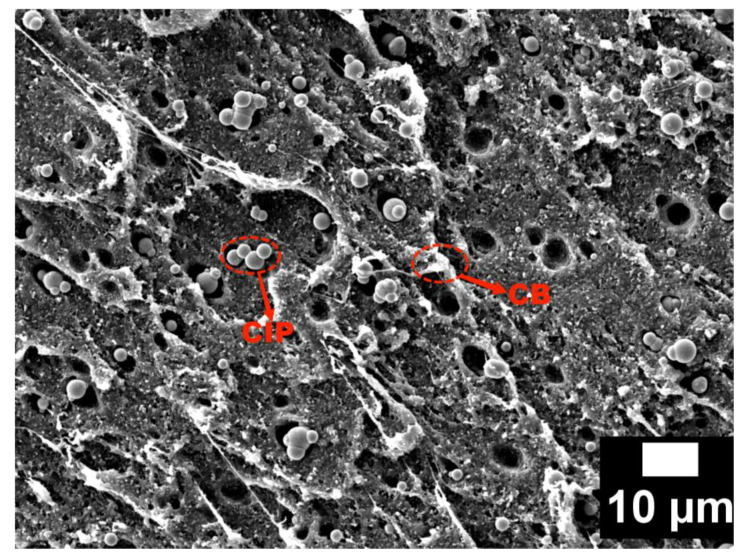
SEM micrographs of the CB20-CIP30 composite.

Figure 8 shows the microscopic morphology of the CB20-CIP30 composite coating under SEM (1000×). It can be observed that the CB and CIP particles were generally uniformly distributed in the PLA matrix, and the inclusion and distribution of the particles would affect the electromagnetic properties of PLA. An agglomeration occurred due to the large specific surface area and surface energy of the CB particles. The agglomeration contributed to the formation of conductive networks, thus increasing the conductivity and dielectric constant of the material. Due to the strong activity and high surface energy of the CIP particles, local agglomeration is likely to occur during the melting or molding process, and the uniformity becomes poor, as shown in Figure 8 in red circles. These agglomerated CIP increased the local eddy current loss and reduced the natural resonance. Because of the difference in particle size, the CB particles existed in the spacing among the CIP particles. In addition, due to the poor compatibility of the particles with the matrix, obvious bulges and wrinkles appeared, and abundant heterogeneous interfaces formed, which were beneficial to enhancing the interfacial polarization loss of the composites.

In order to further explore the loss mechanism of the CB20-CIP30 composite, the impedance matching and attenuation constant were analyzed, as shown in the following Equations (3) and (4) [32].

(3) $$Z=\left|\frac{Z_{{}_{in}}}{Z_{0}}\right|\propto 1$$

(4) $$\alpha =\frac{\sqrt{2}\pi f}{c}\sqrt{\left(\mu^{\prime \prime }\varepsilon^{\prime \prime }-\mu^{\prime }\varepsilon^{\prime }\right)+\sqrt{{\left(\mu^{\prime \prime }\varepsilon^{\prime \prime }-\mu^{\prime }\varepsilon^{\prime }\right)}^{2}+{\left(\mu^{\prime \prime }\varepsilon^{\prime }+\mu^{\prime }\varepsilon^{\prime \prime }\right)}^{2}}}$$

**Figure 9:**
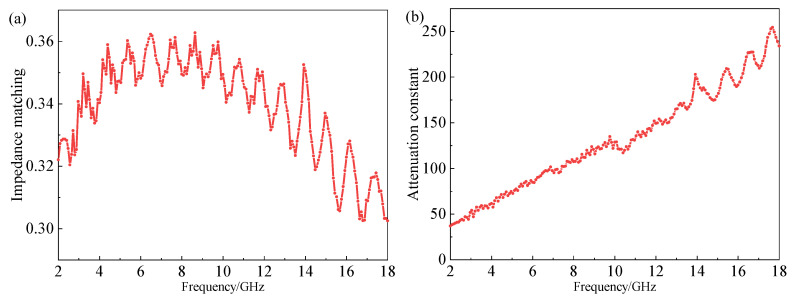
(**a**) Impedance matching and (**b**) attenuation constant of the CB20-CIP30 composite.

Generally, the impedance matching of the absorbing materials should exceed 0.3, and the closer to 1, the better the impedance matching of the material so that the incident wave can enter well into the material, thus reducing the reflection on the surface of the material [33,34]. Figure 9 shows the impedance matching and attenuation constant of the CB20-CIP30 composite. The impedance matching value of the CB20-CIP30 composite was relatively stable, with only small fluctuations, exceeding 0.3 in the range of 2–18 GHz, which indicates that only a small part of the incident wave was reflected at the interface. The attenuation constant showed an upward trend with the increase in frequency; the *α* value increased from 37 to 234, and the loss ability was strong.

From the loss tangent values shown in Figure 3e,f, it can be observed that the dielectric loss tangent value of the CB20-CIP30 composite was much larger than the magnetic loss tangent value, and the loss capacity of the material was mainly dielectric loss, with magnetic loss as a supplement.

**Figure 10:**
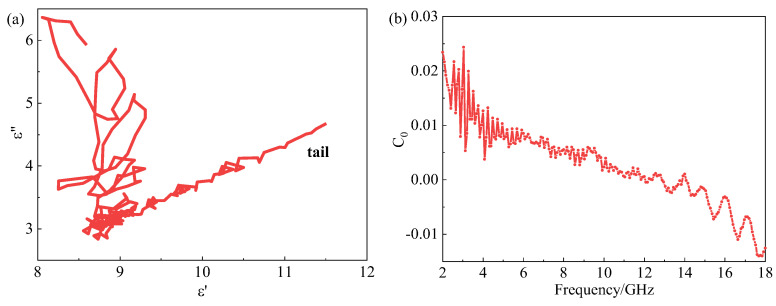
(**a**) Cole-Colo curve, (**b**) $C_{0}$ value of the CB20-CIP30 composite.

Dielectric loss mainly includes conduction loss and polarization relaxation loss (electron polarization, ion polarization, dipole polarization and interface polarization), among which electron polarization and ion polarization usually occur in the THz and PHz range, so they are not considered in the GHz range. The dielectric loss can be explained by the Debye medium theory. The Cole-Cole curve, which is the relationship of $\varepsilon^{\prime }$ and $\varepsilon^{\prime \prime }$, can be expressed as shown in the following Equation (5) [35,36,37]:

(5) $${\left(\varepsilon^{\prime }-\frac{\varepsilon_{s}+\varepsilon_{\infty }}{2}\right)}^{2}+{\left(\varepsilon^{\prime \prime }\right)}^{2}={\left(\frac{\varepsilon_{s}-\varepsilon_{\infty }}{2}\right)}^{2}$$

where $\varepsilon_{s}$ and $\varepsilon_{\infty }$ are the static dielectric constant and the optical dielectric constant, respectively. It can be seen from Equation (5) that the curves of $\varepsilon^{\prime }$ and $\varepsilon^{\prime \prime }$ are a single semicircle, usually defined as a Cole-Cole semicircle, and each semicircle corresponds to one Debye relaxation process, while the appearance of the upward tail indicates that conductive loss contributes to the dielectric loss [38]. The $\varepsilon^{\prime }-\varepsilon^{\prime \prime }$ curves of the CB20-CIP30 composite are shown in Figure 10a. It can be seen that there were several semicircles with overlapping parts, indicating that multiple polarization relaxations occurred. The appearance of a straight line at the tail indicates the contribution of conductive loss was enhanced, and the appearance of irregular semicircles indicates that other loss mechanisms existed, such as interface polarization and dipole polarization, etc. [27,39].

It was reported that magnetic losses mainly come from domain wall resonance, hysteresis loss, eddy current loss, natural resonance and exchange resonance, among which the domain wall resonance mainly appears in the MHz region. As a soft magnetic material, CIP hysteresis loss is so low that it can be ignored [23,40]. Therefore, eddy current loss, natural resonance and exchange resonance are the three important elements that could be responsible for the magnetic loss. The eddy current loss is particularly associated with thickness d and conductivity σ, as shown in the following Equation (6) [31,41]:

(6) $$\mu^{\prime \prime }\approx \frac{2\pi \mu_{0}{\left(\mu^{\prime }\right)}^{2}\sigma d^{2}f}{3}$$

where $\mu_{0}$ is the permeability of the vacuum. The value of $C_{0}$ is commonly used to express the effect of eddy current on magnetic loss, as shown in the following Equation (7) [42].

(7) $$C_{0}=\mu^{\prime \prime }{\left(\mu^{\prime }\right)}^{-2}f^{-1}\approx \frac{2\pi \mu_{0}\sigma d^{2}}{3}$$

If the magnetic loss only originates from the eddy current losses, the value of $C_{0}$ should remain constant over the entire frequency range.

However, the value of $C_{0}$ was not constant in the 2–18 GHz range, indicating eddy current loss was not the only magnetic loss mechanism, as shown in Figure 10b. Therefore, the magnetic loss of the CB20-CIP30 composite was the result of the synergistic effect of the natural resonance, exchange resonance and eddy current loss.

## 4. Conclusions

The application of 3D printing technology to fabricate a multi-component metamaterial absorber with integrated structure and function is an important development direction. The premise was to prepare 3D printing materials with excellent absorbing properties.

The CB-CIP/PLA composite filament was successfully prepared by high-energy mixing and the twin-screw melt extrusion process; then, FDM was used to prepare lightweight, broadband and strong absorbing materials, which had both dielectric and magnetic losses. The absorbing mechanism was analyzed using the electromagnetic parameters. In summary, the 20 wt.% CB and 30 wt.% CIP composites had the highest absorbing properties, which were mainly due to good impedance matching and the synergy of multiple loss mechanisms. According to the calculation results of the transmission line theory, the minimum *RL* value was −51.10 dB (at 16.24 GHz) when the coating thickness was 1.6 mm. When the coating thickness was 1.7 mm, the effective absorption bandwidth reached 5.04 GHz (12.96–18 GHz), covering 90% of the Ku-band (12.4–18 GHz).

Most importantly, here, the preparation method was fairly simple, inexpensive and efficient; hence, the as-prepared CB-CIP/PLA composite filament is a promising candidate as an FDM raw material for a metamaterial absorber. Therefore, the next research task will be to carry out the structural design of the metamaterial absorber based on this composite filament and realize the structure–function integrated metamaterials preparation.

## Figures and Tables

**Table 1:** Ratio of CB and CIP contents refers to PLA.

Samples	Radio Refered to PLA	CB5-CIP10	CB10-CIP10	CB20-CIP10	CB5-CIP30	CB10-CIP30	CB20-CIP30	CB5-CIP50	CB10-CIP50	CB20-CIP50
CB	wt.% (vol.%)	5 (2.78)	10 (5.56)	20 (11.12)	5 (2.78)	10 (5.56)	20 (11.12)	5%	10 (5.56)	20 (11.12)
CIP	wt.% (vol.%)	10 (1.27)	10 (1.27)	10 (1.27)	30 (3.81)	30 (3.81)	30 (3.81)	50 (6.35)	50 (6.35)	50 (6.35)

**Table 2:** Relation between *RL* and percentage absorption.

RL/dB	% of Electromagnetic Energy Absorbed
<−5	>70
<−10	>90
<−15	>96.8
<−20	>99
<−40	>99.99

**Table 3:** Comparison of absorbing properties with the reported CIP composite materials.

Absorber	Matrix	EAB/GHz	Minimum *RL*/dB	Matching Thickness/mm	Reference
CIP/GNs	PU	7.10	−30.10	1.0	[25]
CIP/PDA@MWCNTs	Paraffin	9.68	−30.69	1.5	[26]
CIP@SiO_2_@MnZnFeO	Paraffin	7.12	−44.24	2.0	[27]
CNT/PVA/CIP	Paraffin	6.30	−50.00	3.0	[28]
RGO/CIP	PMMA	3.41	−54.40	2.1	[24]
nmCIP/CB	PU	3.60	−25.80	2.0	[29]
CIPs/CF	Epoxy	1.50	−13.80	2.0	[30]
CIPs/ZnO/RGO	Paraffin	0.41	−45.57	4.0	[31]
RGO/CIP	PLA	2.92	−27.25	3.0	[10]
CB/CIP	PLA	5.04	−51.10	1.6	This work

## References

[1] Meng X., Zhang J., Xiong L., Zhang T., Zhao H., Zhong B., Xia L., Huang X., Wen G. (2018). In-Situ Growth of Fe/Fe_3_O_4_/C Hierarchical Architectures with Wide-Band Electromagnetic Wave Absorption. Ceram. Int..

[2] Qin M., Zhang L., Wu H. (2022). Dielectric Loss Mechanism in Electromagnetic Wave Absorbing Materials. Adv. Sci..

[3] Liang X., Liu W., Cheng Y., Lv J., Dai S., Tang D., Zhang B., Ji G. (2018). Review: Recent Process in the Design of Carbon-Based Nanostructures with Optimized Electromagnetic Properties. J. Alloys Compd..

[4] Min C., Yuxi D., Kang X., Xiaofeng H., Jiayu H., Xi Y. (2020). Research Progress of New Carbon Based Magnetic Composite Electromagnetic Waveabsorbing Materials. Acta Mater. Compos. Sin..

[5] Yang W., Jiang B., Che S., Yan L., Li Z., Li Y. (2021). Research Progress on Carbon-Based Materials for Electromagnetic Wave Absorption and the Related Mechanisms. New Carbon Mater..

[6] Sista K.S., Dwarapudi S., Kumar D., Sinha G.R., Moon A.P. (2021). Carbonyl Iron Powders as Absorption Material for Microwave Interference Shielding: A Review. J. Alloys Compd..

[7] Wei H., Zhang Z., Zhou L., Heidarshenas B., Zhang C., Xia J., Zhi L., Shen G., Wu H. (2020). Influence of Heat Treatment on the Microwave Absorption Properties of Flaky Carbonyl Iron Powder. Int. J. Lightweight Mater. Manuf..

[8] Liu T., Zhou L., Zheng D., Xu Y. (2017). Absorption Property of C@Cips Composites by the Mechanical Milling Process. Appl. Phys. A.

[9] Qing Y.C., Zhou W.C., Jia S., Luo F., Zhu D.M. (2010). Electromagnetic and Microwave Absorption Properties of Carbonyl Iron and Carbon Fiber Filled Epoxy/Silicone Resin Coatings. Appl. Phys. A.

[10] Xicong Y., Bin O., Chao Y., Zhenglang H., Enyi H., Haihua W. (2022). Preparation of Graphene_Carbonyl Iron Powder Wire and Analysis of its Wave Absorption Performance. Acta Mater. Compos. Sin..

[11] Chai L., Wang Y., Zhou N., Du Y., Zeng X., Zhou S., He Q., Wu G. (2021). In-Situ Growth of Core-Shell Znfe_2_O_4_ @ Porous Hollow Carbon Microspheres as an Efficient Microwave Absorber. J. Colloid Interf. Sci..

[12] Green M., Chen X. (2019). Recent Progress of Nanomaterials for Microwave Absorption. J. Mater..

[13] Lei L., Yao Z., Zhou J., Wei B., Fan H. (2020). 3D Printing of Carbon Black/Polypropylene Composites with Excellent Microwave Absorption Performance. Compos. Sci. Technol..

[14] Li W., Liu Y., Guo F., Du Y., Chen Y. (2021). Self-Assembly Sandwich-Like Fe, Co, Or Ni Nanoparticles/Reduced Graphene Oxide Composites with Excellent Microwave Absorption Performance. Appl. Surf. Sci..

[15] Qu Z., Wang Y., Wang W., Yu D. (2021). Robust Magnetic and Electromagnetic Wave Absorption Performance of Reduced Graphene Oxide Loaded Magnetic Metal Nanoparticle Composites. Adv. Powder Technol..

[16] Jin C., Wu Z., Zhang R., Qian X., Xu H., Che R. (2021). 1D Electromagnetic-Gradient Hierarchical Carbon Microtube Via Coaxial Electrospinning Design for Enhanced Microwave Absorption. Acs Appl. Mater. Inter..

[17] Tho P.T., Xuan C.T.A., Tran N., Tuan N.Q., Jeong W.H., Kim S.W., Quang D.T., Nguyen V.D., Bach T.N., Thanh T.D. (2022). Ultra-Wide Effective Absorption Bandwidth of Cu, Co, and Ti Co-Doped Srfe_12_O_19_ Hexaferrite. Ceram. Int..

[18] Yu J., Li Y., Xu X., Duan G., Li Y., Zhou W. (2021). Rambutan-Like Nb_2_O_5_@Shcs Microspheres for Improved Microwave Absorption Performance. Compos. Commun..

[19] Zhang W., Bie S., Chen H., Lu Y., Jiang J. (2014). Electromagnetic and Microwave Absorption Properties of Carbonyl Iron/Mno_2_ Composite. J. Magn. Magn. Mater..

[20] Zhao B., Shao G., Fan B., Zhao W., Zhang R. (2015). Investigation of the Electromagnetic Absorption Properties of Ni@Tio_2_ and Ni@Sio_2_ Composite Microspheres with Core-Shell Structure. Phys. Chem. Chem. Phys..

[21] Ruiz-Perez F., López-Estrada S.M., Tolentino-Hernández R.V., Caballero-Briones F. (2022). Carbon-Based Radar Absorbing Materials: A Critical Review. J. Sci. Adv. Mater. Devices.

[22] Ning M., Li J., Kuang B., Wang C., Su D., Zhao Y., Jin H., Cao M. (2018). One-Step Fabrication of N-Doped Cnts Encapsulating M Nanoparticles (M = Fe, Co, Ni) for Efficient Microwave Absorption. Appl. Surf. Sci..

[23] Liu P., Ng V.M.H., Yao Z., Zhou J., Lei Y., Yang Z., Lv H., Kong L.B. (2017). Facile Synthesis and Hierarchical Assembly of Flowerlike Nio Structures with Enhanced Dielectric and Microwave Absorption Properties. Acs Appl. Mater. Inter..

[24] Zuo Y., Yao Z., Lin H., Zhou J., Lu J., Ding J. (2019). Digital Light Processing 3D Printing of Graphene/Carbonyl Iron/Polymethyl Methacrylate Nanocomposites for Efficient Microwave Absorption. Compos. Part B Eng..

[25] Duan Y., Liu Y., Cui Y., Ma G., Tongmin W. (2018). Graphene to Tune Microwave Absorption Frequencies and Enhance Absorption Properties of Carbonyl Iron/Polyurethane Coating. Prog. Org. Coat..

[26] Zheng J., Zhou Z., Zhu L., Chen Q., Hong M., Fu H. (2022). Room Temperature Self-Healing Cip/Pda/Mwcnts Composites Based On Imine Reversible Covalent Bond as Microwave Absorber. React. Funct. Polym..

[27] Chen Q., Li L., Wang Z., Ge Y., Zhou C., Yi J. (2019). Synthesis and Enhanced Microwave Absorption Performance of Cip@ Sio_2_@Mn_0.6_Zn_0.4_Fe_2_O_4_ Ferrite Composites. J. Alloys Compd..

[28] Peng X., Zhang Q., Xie Z. (2020). Preparation and Wave Absorption of Carbon Nanotube/Polyvinyl Alcohol/Carbonyl Iron Powder Composites. Carbon Technol..

[29] Li X., Zhang Y., Chen J., Duan Y., Wu G., Ma G. (2013). Composite Coatings Reinforced with Carbonyl Iron Nanoparticles: Preparation and Microwave Absorbing Properties. Mater. Technol..

[30] Hamed Salimkhani P.P.A.B. (2016). Electrophoretic Deposition of Spherical Carbonyl Iron Particles on Carbon Fibers as a Microwave Absorbent Composite. Surf. Interfaces.

[31] Yin P., Zhang L., Wang J., Feng X., Wang K., Rao H., Wang Y., Dai J. (2019). Low Frequency Microwave Absorption Property of Cips/Zno/Graphene Ternary Hybrid Prepared Via Facile High-Energy Ball Milling. Powder Technol..

[32] Li N., Huang G., Li Y., Xiao H., Feng Q., Hu N., Fu S. (2017). Enhanced Microwave Absorption Performance of Coated Carbon Nanotubes by Optimizing the Fe_3_O_4_ Nanocoating Structure. Acs Appl. Mater. Inter..

[33] Liu L., He N., Sun J., Hu P., He R., Cheng J., Tian W., Tong G. (2018). Tailoring Impedance Match and Enhancing Microwave Absorption of Fe_3_O_4_/Bi_24_Fe_2_O_39_/Bi Hollow Porous Microrods by Controlling their Composition. Prog. Nat. Sci. Mater. Int..

[34] Wei B., Zhou J., Yao Z., Haidry A.A., Qian K., Lin H., Guo X., Chen W. (2020). Excellent Microwave Absorption Property of Nano-Ni Coated Hollow Silicon Carbide Core-Shell Spheres. Appl. Surf. Sci..

[35] Jian X., Wu B., Wei Y., Dou S.X., Wang X., He W., Mahmood N. (2016). Facile Synthesis of Fe_3_O_4_/Gcs Composites and their Enhanced Microwave Absorption Properties. Acs Appl. Mater. Inter..

[36] Li Y., Meng F., Mei Y., Wang H., Guo Y., Wang Y., Peng F., Huang F., Zhou Z. (2020). Electrospun Generation of Ti_3_C_2_Tx Mxene@Graphene Oxide Hybrid Aerogel Microspheres for Tunable High-Performance Microwave Absorption. Chem. Eng. J..

[37] Shi Y., Yu L., Li K., Li S., Dong Y., Zhu Y., Fu Y., Meng F. (2020). Well-Matched Impedance of Polypyrrole-Loaded Cotton Non-Woven Fabric/Polydimethylsiloxane Composite for Extraordinary Microwave Absorption. Compos. Sci. Technol..

[38] Hou T., Jia Z., Wang B., Li H., Liu X., Bi L., Wu G. (2021). Mxene-Based Accordion 2D Hybrid Structure with Co_9_S_8_/C/Ti_3_C_2_Tx as Efficient Electromagnetic Wave Absorber. Chem. Eng. J..

[39] Liu D., Du Y., Xu P., Liu N., Wang Y., Zhao H., Cui L., Han X. (2019). Waxberry-Like Hierarchical Ni@C Microspheres with High-Performance Microwave Absorption. J. Mater. Chem. C.

[40] Liu Y., Yao Z., Zhou J., Jin L., Wei B., He X. (2022). Facile Synthesis of Mof-Derived Concave Cube Nanocomposite by Self-Templated Toward Lightweight and Wideband Microwave Absorption. Carbon.

[41] Zhang K., Chen J., Yue S., Zhang H., Meng C., Wang J. (2020). Facile Synthesis of Core-Shell Ci/Sio_2_ Decorated Rgo Sheets Composite for Excellent Electromagnetic Wave Absorption Performance Covering the Whole X-Band. Compos. Part A Appl. Sci. Manuf..

[42] Long Q., Xu Z., Xiao H., Xie K. (2018). A Facile Synthesis of a Cobalt Nanoparticle-Graphene Nanocomposite with High-Performance and Triple-Band Electromagnetic Wave Absorption Properties. RSC Adv..

